# Phase‐Pole‐Free Images and Smooth Coil Sensitivity Maps by Regularized Nonlinear Inversion

**DOI:** 10.1002/mrm.70333

**Published:** 2026-03-12

**Authors:** Moritz Blumenthal, Martin Uecker

**Affiliations:** ^1^ Institute of Biomedical Imaging Graz University of Technology Graz Austria; ^2^ Institute for Diagnostic and Interventional Radiology University Medical Center Göttingen Göttingen Germany; ^3^ DZHK (German Centre for Cardiovascular Research) Germany; ^4^ BioTechMed‐Graz Graz Austria

**Keywords:** image reconstruction, MRI, non‐linear inverse problems, parallel imaging, phase singularity

## Abstract

**Purpose:**

Phase singularities are a common problem in image reconstruction with auto‐calibrated sensitivities due to an inherent ambiguity of the estimation problem. The purpose of this work is to develop a method for detecting and correcting phase poles in non‐linear inverse (NLINV) reconstruction of MR images and coil sensitivity maps.

**Methods:**

Phase poles are detected in individual coil sensitivity maps by computing the curl in each pixel. A weighted average of the curl in each coil is computed to detect phase poles. Phase pole detection and correction is then integrated into the iteratively regularized Gauss‐Newton method of the NLINV algorithm. In addition, we demonstrate the algorithm can also remove phase poles in ESPIRiT coil sensitivity maps. Phase pole correction is evaluated for reconstruction of accelerated Cartesian MPRAGE data of the brain and interactive radial real‐time MRI of the human heart.

**Results:**

For both applications, phase pole correction can be used for estimation of coil sensitivity profiles free from singularities. For NLINV, we demonstrate reliable and efficient estimation even from very small (7×7) auto‐calibration (AC) regions.

**Conclusion:**

With the proposed method, phase poles can be reliably removed in reconstructions and coil sensitivities.

## 1 Introduction

MR images are inherently complex‐valued. While most clinical applications use the magnitude images for diagnosis, the phase carries information about the local magnetic field experienced by the spins during the acquisition. This includes contributions from local tissue susceptibility, flow, and chemical shift. Extracting this information forms the basis of many advanced MRI applications.

In practice, the MR signal is usually measured by phased array coils [1], such that the complex‐valued image is multiplied with the complex‐valued coil sensitivity profiles. To recover the original image, the coil sensitivity profiles need to be known absolutely. However, from the measured data itself, only relative coil sensitivity profiles can be estimated [2, 3, 4, 5, 6]. When absolute coil sensitivity profiles are not available, a channel with relatively homogeneous sensitivity can arbitrarily be chosen as a reference, or coils can be normalized to have a root‐sum‐of‐squares (RSS) of one such that coil combination results in a complex‐valued image with a magnitude corresponding to the conventional RSS coil combination [1]. The latter does not define a phase. Normalization techniques to select a phase include selection of an arbitrary coil as reference [5, 6, 7], the construction of virtual reference coils [8, 9, 10, 11] or normalization such that the image is roughly real‐valued by shifting the phase into the coil sensitivities [12, 13]. While the latter complicates processing for applications which require the image phase, the former effectively sets the image phase to the phase of the respective coil image. If this coil image contains phase poles, this leads to phase poles, i.e., singularities, in the images (c.f., Figure 6), which are also called open‐ended fringe lines in the phase‐unwrapping literature [14, 15, 16] and could be misinterpreted as microhemorrhages in susceptibility weighted imaging [17].

In the SENSE [18, 19] formulation of parallel imaging, coil sensitivity profiles are used to unfold aliased images. Nowadays, SENSE reconstruction is often formulated as a linear inverse problem. In case of $\ell_{2}$‐regularization, the reconstruction is invariant to the phase of the images. However, if SENSE is combined with compressed sensing [20], the reconstruction may be sensitive to the phase of the images such as for $\ell_{1}$‐Wavelet regularization [21]. To overcome phase problems in compressed sensing reconstructions, regularization terms that are explicitly designed to be phase‐invariant have been proposed [22, 23, 24]. k‐Space‐based parallel imaging reconstructions [25, 26, 27, 28] effectively work with the k‐space of the respective coil images and are hence invariant to the phase ambiguity, as selection of the final image phase is postponed to the coil combination step. Nevertheless, SENSE‐based methods simplify the use of image‐based regularization terms or physics‐based models [29].

NLINV [30] is a parallel imaging reconstruction method which jointly estimates the image and the coil sensitivity profiles by regularized non‐linear inversion. NLINV penalizes the Sobolev norm of the coil sensitivities, which leads to smooth coil sensitivities. Due to this smoothness, coil sensitivities can be efficiently estimated on a low‐resolution grid and interpolated to target resolution [31] without the danger of introducing artifacts due to non‐smooth phase variations. As NLINV can directly work with non‐Cartesian data, it can be used for coil sensitivity estimation in this setting, and is especially useful in 3D where computational cost is an issue [32, 33, 34, 35, 36, 37, 38, 39, 40]. Various extensions to NLINV have been developed [41, 42, 43], including for real‐time MRI [30, 44] and deep‐learning‐based reconstruction [45, 46], it has been adapted to various specific applications [47, 48, 49, 50, 51, 52, 53, 54, 55, 56, 57], and used in clinical research [58, 59, 60, 61, 62, 63, 64]. Despite its successful application in many challenging scenarios, NLINV still suffers from one problem that prevents its use in routine applications. NLINV solves a non‐linear optimization using an iterative method which can get trapped in local minima. This problem seems directly related to the phase ambiguity described above, as the local minima observed in practice always contain phase poles in the image and conjugate phase poles in the coil sensitivity profiles. This then leads to black holes in regularized reconstructions [43, 65, 66]. If a body coil as reference is available, the non‐linear inversion can be initialized to deliver phase‐pole‐free images [66], but a body coil reference is not always available. Extending NLINV to ENLIVE [43] provides artifact free magnitude images by simultaneously reconstructing multiple images corresponding to multiple sets of coil sensitivities similar to ESPIRiT [5]. However, the image phase may still contain phase poles.

In this work, we present a method to detect convergence of NLINV to a local minimum with phase poles by detecting phase poles and correcting them by a global optimization step, similar to a re‐initialization of the algorithm. In this global optimization step the phase pole in the image is removed by multiplying a conjugate pole to the image while multiplying the pole itself on the coil sensitivity profiles. While the method is not restricted to NLINV, it is especially well‐suited for NLINV since slightly misaligned corrections will be refined in consecutive iterations. We evaluate NLINV with phase pole correction on Cartesian brain data and on radial, interactive real‐time MRI data. Moreover, we show that NLINV can reliably and efficiently estimate coil sensitivity profiles free from singularities from very small (7×7) auto‐calibration (AC) regions.

## 2 Methods

The MR signal $s(k)\in {\mathbb{C}}^{N_{C}}$ measured by a phased‐array coil with $N_{C}$ coil elements is the Fourier transform of the product of the coil sensitivity $c(r)\in {\mathbb{C}}^{N_{C}}$ and the image ρ(r)∈ℂ at position $r\in {\mathbb{R}}^{d}$, that is,

(1) $$\begin{array}{l} s(k)=\int_{{\mathbb{R}}^{d}}d^{d}r\;c(r)\rho (r)e^{-i2\pi k\cdot r}\;. \end{array}$$

Here, phase due to the transmit coil is absorbed into c such that the phase ϕ(r) of the image ρ(r) is the time‐integrated local Larmor frequency, that is, $\phi (r)=\int_{0}^{t_{E}}\omega_{L}(r,t)dt$. As only the product of image and coil sensitivity is measured, both cannot be uniquely determined from the data but only up to a complex scaling function ϑ(r)∈ℂ∖{0}, that is, if ρ and c are consistent with the data, then $\rho^{\prime }=\vartheta^{-1}\rho$ and $c^{\prime }=\vartheta c$ are consistent, too. To break this ambiguity, a normalization of the coil sensitivities is required.

### 2.1 Phase Poles and Singularities in MRI

Let $\Psi (r)=A(r)e^{-i\phi (r)}$ be a complex field, such as an image or coil sensitivities, with amplitude A(r) and phase ϕ(r). A phase pole (or phase singularity) is a point $r_{0}$ with undefined phase ϕ(r) further characterized by the condition that a closed loop curve integral of the phase gradient ∇ϕ along a curve $𝒞\in {\mathbb{R}}^{d}$ around the point is not zero, that is,

(2) $$\begin{array}{l} 0\neq S_{𝒞}=\frac{1}{2\pi }\oint_{𝒞}\nabla \phi (s)ds. \end{array}$$

**FIGURE 1:**
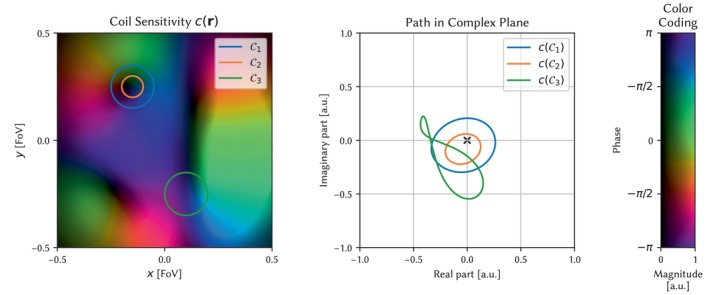
Example for a phase singularity in the coil sensitivity profile. The curves ${𝒞}_{1}$ and ${𝒞}_{2}$ enclose the same phase singularity such that their image $c({𝒞}_{1})$ and $c({𝒞}_{2})$ wind once around the origin in the complex plane. The curve ${𝒞}_{3}$ does not enclose the singularity and therefore has a winding number of zero.

By interpreting the complex number Ψ(r) as a point in the complex plane, the integral $S_{𝒞}$ corresponds to the winding number of the curve Ψ(𝒞)∈ℂ [67]. For a smooth field Ψ(r), a phase pole in $r_{0}$ implies a vanishing amplitude $A(r_{0})=0$. An example for a phase pole in a coil sensitivity profile and lifting a curve 𝒞 to the complex plane is shown in Figure 1.

It should be noted that the phase of the image ρ(r) originates from the time‐integrated local Larmor frequency, that is, $\phi (r)=\int_{0}^{t_{E}}\omega_{L}(r,t)dt$ and is therefore well‐defined and single‐valued in regions with non‐vanishing signal. Hence, the true, that is, noise‐free and non‐discretized, image should not have phase singularities. In contrast, the phase of the coil sensitivities c(r) can have phase singularities [12]. Recalling the principle of reciprocity, the complex valued coil sensitivity c(r) corresponds to the transversal components of the magnetic field ℬ(r) generated by the coil, that is, $c(r)\propto {ℬ}_{x}-i{ℬ}_{y}$ [68]. An example for a phase singularity is the field of a magnetic dipole $m=m{ê}_{z}$ in the origin. For z≠0, the coil sensitivity profile in polar coordinates is given by $c(r,\phi ,z)=|c(r,z)|e^{i\phi }$. It should be noted that even if the physical coil sensitivities have no phase singularities, coil compression [8, 69, 70] techniques can introduce phase singularities in virtual coils (c.f., Figure 6).

### 2.2 NLINV Reconstruction

NLINV [4] is a parallel imaging reconstruction technique which jointly estimates the image ρ and the coil sensitivity maps c. It formulates the reconstruction as a non‐linear inverse problem with the forward model F(ρ,c)=𝒫ℱ(c⊙ρ), where ⊙ is the Hadamard product, that is, point wise multiplication, ℱ is the Fourier transform and 𝒫 is the projection to the Cartesian or non‐Cartesian sampling pattern. The regularized reconstruction can then be formulated as the non‐linear optimization problem

(3) $$\begin{array}{l} (\rho ,c)=\underset{\rho ,c}{\arg\min}\|y-F(\rho ,c){\|}_{2}^{2}+\alpha \|\rho {\|}_{2}^{2}+\alpha \|Wc{\|}_{2}^{2}, \end{array}$$

where the $\ell_{2}$‐norm of the image and the Sobolev norm of the coil sensitivities are penalized. Here, the Sobolev norm is represented as a weighted $\ell_{2}$‐norm with a weighting matrix W that penalizes the high frequency components of the coil sensitivities. For efficient computation, the optimization is preconditioned by introducing

(4) $$\begin{array}{ll} \tilde{c} & =W^{-1}c\;\text{and}\;\tilde{F}(\rho ,\tilde{c})=F(\rho ,W\tilde{c}), \end{array}$$

such that (3) is equivalent to

(5) $$\begin{array}{l} (\rho ,\tilde{c})=\underset{\rho ,\tilde{c}}{\arg\min}\|y-\tilde{F}(\rho ,\tilde{c}){\|}_{2}^{2}+\alpha \|\rho {\|}_{2}^{2}+\alpha \|\tilde{c}{\|}_{2}^{2}\;. \end{array}$$

This optimization problem is solved using the iteratively regularized Gauss‐Newton method (IRGNM), where the regularization parameter α is chosen for every iteration k such that $\alpha^{\left(k\right)}$ exponentially decays from one to $\alpha_{\min}$. In each Gauss‐Newton step, the non‐linear forward model $\tilde{F}$ is linearized around the current estimate $\left(\rho^{\left(k\right)},{\tilde{c}}^{\left(k\right)}\right)$. In case of $\ell_{2}$‐regularization, the inner problem is quadratic problem and can be solved using a conjugate gradient method with the corresponding regularization parameter $\alpha^{\left(k\right)}$. To favor real‐valued images, NLINV is initialized by setting the image to constant one and the coil sensitivities to zero. The k‐space data is normalized such that ${\left\|y\right\|}_{2}=100$ which is empirically found to yield good results for 2D imaging. The final reconstruction is usually rescaled to yield the typical RSS scaling, that is,

(6) $$\begin{array}{ll} \rho & \leftarrow \rho \cdot \sqrt{\overset{N_{C}}{\underset{i=1}{\sum }}|c_{i}{|}^{2}}\;\text{and}\;c\;\leftarrow c\cdot \frac{1}{\sqrt{\sum_{i=1}^{N_{C}}|c_{i}{|}^{2}}}. \end{array}$$

Since the objective function in (5) is non‐convex, the Gauss‐Newton method can converge to local minima. One class of local minima are solutions with a phase singularity in the image ρ(r). Even though these phase singularities are not physically meaningful, they can occur when they are compensated by a conjugate singularity in the coil sensitivity due to the inherent ambiguity in the problem. Due to the smoothness penalty of the coil sensitivities, the magnitude of the coil sensitivity must be zero at the phase singularity. In a reconstruction with regularization, this leads to a black hole, that is, vanishing signal in the reconstructed image [43, 65, 66].

### 2.3 Phase‐Pole Correction in NLINV

To avoid phase pole, we propose to detect and remove artificial poles during the iterative reconstruction. If a pole is present at location $r_{0}$ in the image, there will be conjugate poles in the coil sensitivity profiles. The pole can then be removed by multiplying the image with the inverse pole and the coil sensitivities with the pole. For this, we use the phase vortex $\vartheta_{r_{0}}^{\pm }(r)$, defined by

(7) $$\begin{array}{l} \vartheta_{r_{0}}^{\pm }(r)=\frac{{\left(r-r_{0}\right)}_{x}\pm i{\left(r-r_{0}\right)}_{y}}{\left\|r-r_{0}\right\|}=e^{\pm i\phi (r-r_{0})}, \end{array}$$

where the phase ϕ corresponds to the phase in polar coordinates with respect to the point $r_{0}$. As NLINV does not work directly with the coil sensitivities c but with the preconditioned coil sensitivities $\tilde{c}$, the correction is applied on c and transformed to $\tilde{c}$ by means of a regularized pseudo inverse $W^{+}$, that is,

(8) $$\begin{array}{ll} \rho^{\left(k\right)} & \leftarrow \vartheta_{r_{0}}^{\pm }⊙\rho^{\left(k\right)}\;;\;{\tilde{c}}^{\left(k\right)}\;\leftarrow W^{+}\left[\vartheta_{r_{0}}^{\mp }⊙W{\tilde{c}}^{\left(k\right)}\right]\;. \end{array}$$

Multiple poles can be corrected simultaneously by using the product of the corresponding vortices.

Phase poles can be detected by computing the winding number $S_{𝒞}$ in (2). The integral can be discretized by splitting the curve 𝒞 into N segments ${𝒞}_{i}$ connecting the points $r_{i}$ and $r_{i+1}$ with $r_{N}=r_{0}$ on the discrete grid, that is,

(9) $$\begin{array}{ll} S_{𝒞} & =\frac{1}{2\pi }\oint_{𝒞}\nabla \phi (s)ds=\frac{1}{2\pi }\overset{N-1}{\underset{i=0}{\sum }}\int_{{𝒞}_{i}}\nabla \phi (s)ds\\ & \approx \frac{1}{2\pi }\overset{N-1}{\underset{i=0}{\sum }}\text{Arg}\left(\exp[i\left(\phi (r_{i+1})-\phi (r_{i})\right)]\right). \end{array}$$

Here, Arg(·) denotes the principal value of the complex argument in (−π,π] to account for phase wrapping. The integral $\int_{{𝒞}_{i}}\nabla \phi (s)ds$ can be computed exactly by the fundamental theorem of calculus, but the phase at the endpoints may be wrapped such that it is only determined up to multiples of 2π. From all possible solutions, we choose the one in the interval (−π,π]. If the complex field itself happens to be zero at one of the endpoints, the phase is undefined and the winding number cannot be computed reliably. We set it to zero in this case.

**FIGURE 2:**
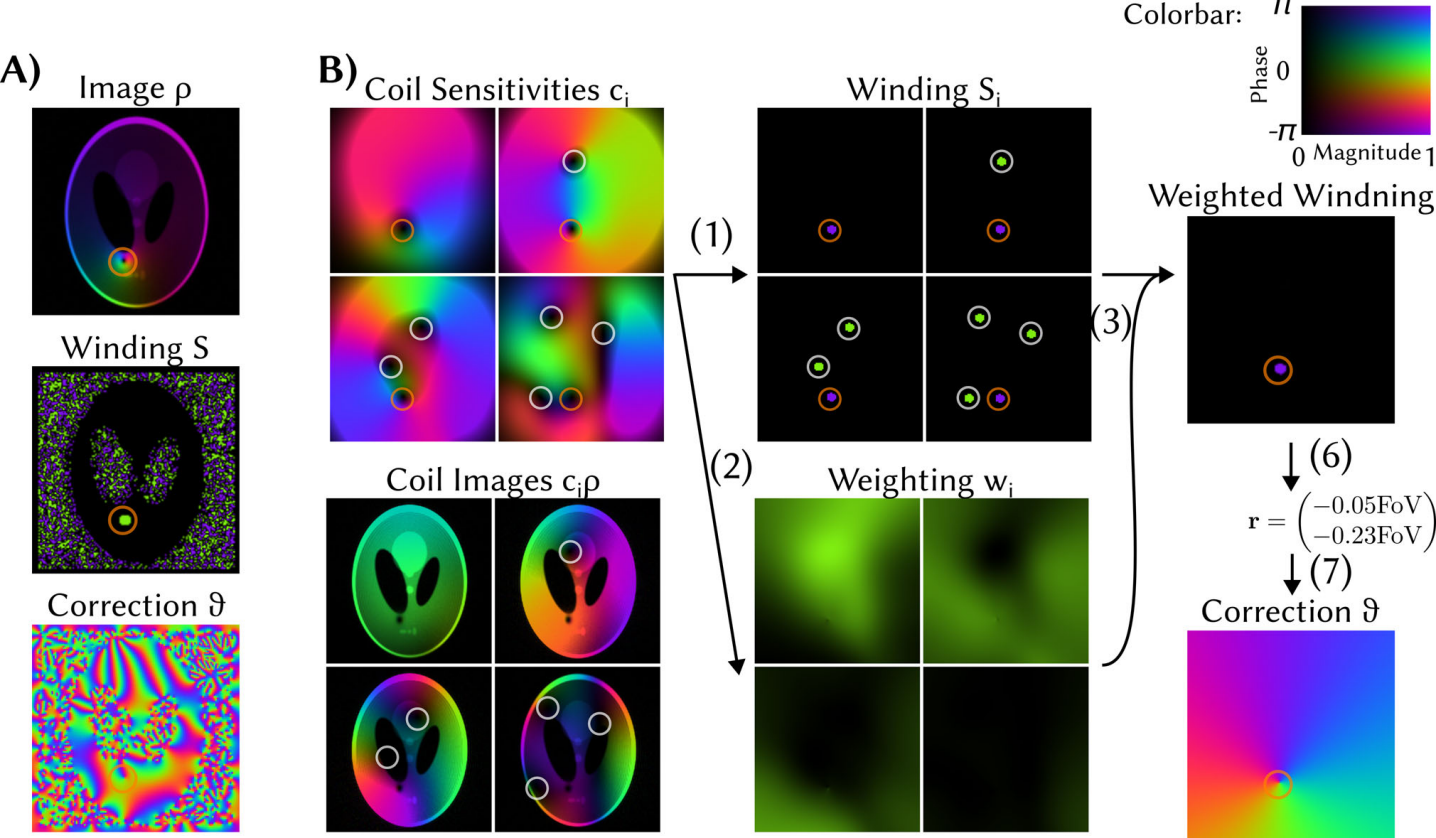
Image‐based (A) and coil‐based‐phase (B) phase pole detection. The image‐based detection is not robust against noise leading to a random correction function. In the coil‐based detection, multiple poles are detected. Those corresponding to true singularities in the sensitivities are marked by white circles and the corresponding poles are also contained in the coil‐images. After taking the weighted average, only the false pole which is present in all coils and in the image is detected, leading to a single factor in the correction function. In this example, we do not show the thresholding step in (4) as it has no visible effect. Furthermore, we skip the morphological closing in step (5), as it would connect all noise poles in the image‐based detection. We refer to Supporting Information: Figure S1 for an example with real data.

Even if the true image ρ(r) does not have phase singularities, the discretized image can show phase singularities due to too coarse discretization or noise [14]. Because the phase pole detection is not robust for images, we propose to detect phase poles in the coil sensitivities $c^{\left(k\right)}$. Assuming a pole in the image, all coil sensitivities should contain the conjugate of the pole as long as a coil does not happen to have a true phase singularity at the same position. As the coil sensitivities are smooth, they do not contain noise corrupting the phase pole detection. Our detection algorithm is based on the following steps which are illustrated in Figure 2 for a schematic example and in Supporting Information: Figure S1 for a real example:

1. For each coil sensitivity $c_{i}$, the winding number $S_{i,𝒞}(r)$ is computed for each pixel r for a curve 𝒞 connecting the grid points $r_{j}\;(j=0\ldots N-1)$ that approximate a small circle around r. The diameter d of the circle must be chosen sufficiently large such that for slightly misaligned poles the detected area in different coils overlaps (c.f., Supporting Information: Figure S1). Too large diameters may lead to overlaps of independent phase poles and spoil spatial resolution of the detection. We empirically choose d=0.05FoV which worked well for high‐ and low‐resolution detection.
2. A weighting function $w_{i}(r)=|c_{i}(r){|}^{2}$ is computed by taking the magnitude squared of the sensitivity maps. This function weights down contribution from physical poles in coil sensitivities as those imply vanishing amplitude.
3. The weighted average $S_{𝒞}(r)=\frac{\sum_{i}w_{i}(r)S_{i,𝒞}(r)}{\sum_{i}w_{i}(r)}$ is computed. As visualized in Figure 2, this step suppresses all detected poles that are true poles in the coil sensitivities.
4. The weighted map $S_{𝒞}(r)$ is thresholded to values with magnitude larger than a threshold t; we use $t=\frac{1}{2}$. This yields a binary map indicating that a voxel is close to a pole in the image. Each of the M connected component of this binary map is considered to correspond to a single pole j=1,…,M.
5. Separated but close connected components are merged by a morphological closing operation with a disk of diameter $d_{\text{closing}}=d$. This step prevents over‐compensation of phase poles by a double correction if one pole is incorrectly interpreted as two close‐by poles (c.f., Supporting Information: Figure S1).
6. The centers of mass $r_{j}$ of the remaining connected components are computed, corresponding to the location of the M respective poles.
7. For each detected pole, the corresponding phase vortex $\vartheta_{r_{j}}^{\pm }(r)$ is computed. Its sign depends on the sign of the weighted average. All phase vortices are multiplied to yield the final correction function $\vartheta (r)=\prod_{j=1}^{M}\vartheta_{r_{j}}^{\pm_{j}}(r)$.
8. A global phase for the correction function is computed such that the influence of the correction on the image is minimized, that is,
(10) $$\begin{array}{ll} \vartheta (r) & \leftarrow \vartheta (r)\cdot \langle \rho ,\vartheta ⊙\rho \rangle /|\langle \rho ,\vartheta ⊙\rho \rangle |. \end{array}$$
  Here, ⟨·,·⟩ denotes the inner product. Intuitively, the inner product averages the complex conjugate of ϑ weighted by the image power, yielding a final correction that changes the image phase as little as possible in high signal regions.

This procedure is designed to detect well‐separated phase poles. If two close poles have the same sign of the winding number, they will be detected as a single pole in step (5). While this has not been observed by us in practice, the correction could be repeated to correct the second pole after the first one has been removed. If two close poles have opposite signs of the winding number, they are likely not detected as poles due to a cancellation in computations of the winding number. However, in practice, we did not observe such cases as close poles with opposite sign are likely to annihilate each other during the iterative reconstruction.

### 2.4 Experiments

All MRI data have been acquired with written informed consent and with approval of the local ethics committee on a Magnetom VIDA 3T (Siemens Healthineers). Phase pole detection and correction was integrated in BART [71, 72] and all reconstructions were performed with BART.

Fully‐sampled 3D Cartesian MPRAGE (TR/TE/TI=2200/2.46/900ms) data of a healthy volunteer were acquired with a 20‐channel head coil. Matrix size was 256×256×208 and resolution $1\times 1\times 0.9\;{\text{mm}}^{3}$. The data was retrospectively undersampled in the $k_{y}-k_{z}$‐plane with a 2×2 regular pattern and fully‐sampled auto‐calibration (AC) regions of size 3×3, 7×7, and 15×15. The data was pre‐whitened [19] and coil‐compressed to 12 virtual coils. After an inverse Fourier transform in the readout direction, the data was reconstructed slice‐wise using NLINV with and without phase pole correction. Twelve Gauss‐Newton steps were performed with a minimal regularization parameter of $\alpha_{\min}=0.001$. Phase pole detection was performed once after eight Gauss‐Newton steps such that the problem is sufficiently converged and four iterations remain for refinement of the reconstruction.

To evaluate the coil sensitivity profiles estimated by NLINV (with and without phase pole correction), we compare them with ESPIRiT [5]. For this, NLINV coil sensitivity profiles were normalized to have an RSS of one as in ESPIRiT. Using the fully‐sampled data, a projection test [5] was performed. For this, the coil images $m={ℱ}^{-1}y$ were computed by an inverse Fourier transform. The coil images were then projected to the subspace spanned by the coil sensitivities by the point‐wise projection operator $P_{c(r)}={\left\|c(r)\right\|}^{-2}c(r)c{\left(r\right)}^{H}$. For well estimated coil sensitivities, the difference to the measured coil images m should be the whitened measurement noise and not contain any image information.

Moreover, for all undersampling patterns, the resulting coil sensitivity profiles were used for an $\ell_{1}$‐Wavlet regularized SENSE reconstruction. As a reference to evaluate these reconstructions, we used the fully‐sampled data, and coil combined images were computed using ESPIRiT coils estimated from a 24×24 AC region.

To demonstrate the efficiency NLINV as a coil sensitivity estimation method, we measured the time to estimate the coil sensitivities with a 15×15 AC region for all 256 slices of the dataset using ESPIRiT, PISCO [6] and NLINV with and without phase pole correction. For all methods, the number of threads used per slice was set to one on an Intel Xeon Gold 6136 CPU. For PISCO, we used the Matlab implementation^1^ with and without interpolation of the final coil sensitivities from a low‐resolution grid. Default parameters were used, except for the threshold of the singular values which, was set to 0.001, and the FFT‐based computation of the calibration matrix, which was disabled due to a too large approximation error on this small AC region, leading to poor coil sensitivities. For NLINV, we estimated coils using the full resolution and on a low‐resolution 48×48 grid.

Moreover, phase pole correction was tested for ESPIRiT coil sensitivities. For this, ESPIRiT coil sensitivities were estimated from the fully‐sampled data using a 24×24 AC region. Since phase poles in ESPIRiT coils are exactly at the same position in all coils, we computed the winding number with a circle of diameter 1 pixel, that is, using the four neighboring pixels forming a square, and the closing operation was skipped. To show the effect of the corrections, coil combination of the fully‐sampled coil images was performed using the original and the corrected ESPIRiT coils.

Phase pole correction was also tested for changing coil sensitivities in interactive real‐time MRI. Radial FLASH data (TR/TE=2.06/0.76ms) was acquired with a turn‐based scheme with 13 spokes per frame and 5 turns [44] for in total 32s, that is, 1200 frames. The imaging slice was changed interactively during the acquisition to trigger a phase poles in the reconstruction. Gradient delays were corrected using RING [73] and the data was compressed to eight virtual coils using geometric coil compression [69, 74]. Real‐time images were reconstructed using real‐time NLINV [30] implemented in BART's streaming framework [74] with and without phase pole correction using six Gauss‐Newton steps, allowing for reconstruction in real time. Phase pole correction was performed after the last step, such that the correction is refined in the following frames.

## 3 Results

**FIGURE 3:**
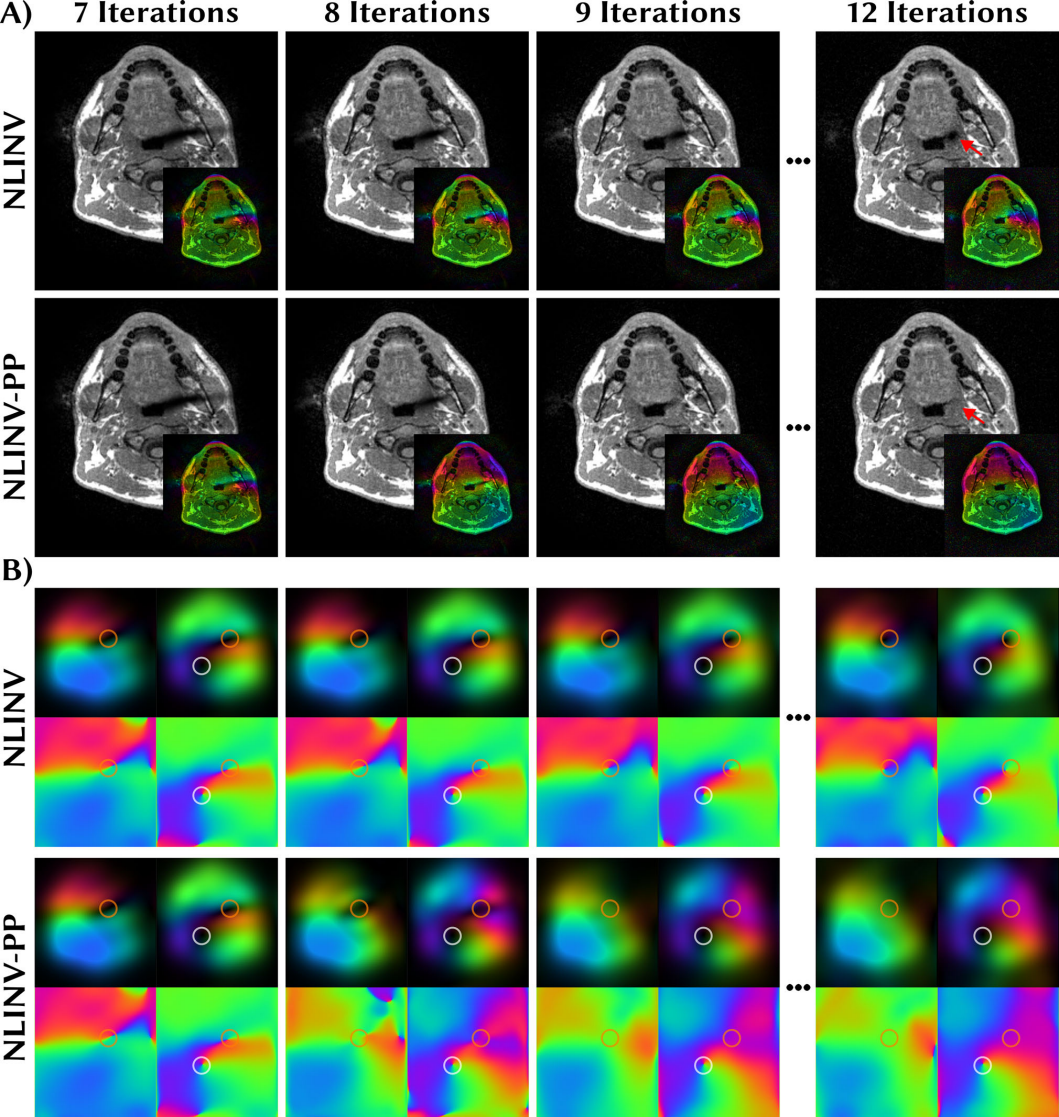
NLINV reconstructions (A) and the first two coil sensitivities (B) with and without phase pole correction. Insets in (A) show the complex image before normalization (6). Coil sensitivities are shown once as complex maps (first row) and once phase only (second row) to highlight the phase poles which are located in the magnitude regions of the coils. After eight iterations, the phase pole is corrected visible by the changed phase in the image and coils. After that the magnitude normalizes over the remaining iterations. In the final reconstruction, the phase pole in the non‐corrected reconstruction leads to a black hole marked by the red arrow. Orange circles mark the position of the phase pole in the coil sensitivities. The white circles mark the position of a true phase pole only present in the 2nd coil sensitivity map. Phase is color‐coded as in Figure 1.

We present the NLINV reconstructions with and without phase pole correction of the retrospectively undersampled data in Figure 3. In the final reconstruction, the phase pole leads to a black hole as the coils are vanishing due to the singularity (marked by red arrow). After eight iterations, the phase pole is detected and corrected, however, the correction does not immediately affect the magnitude of the image. In the phase‐pole corrected reconstruction, the magnitude normalizes after correction over the remaining four Gauss‐Newton steps, leading to a homogeneous image without singularity.

**FIGURE 4:**
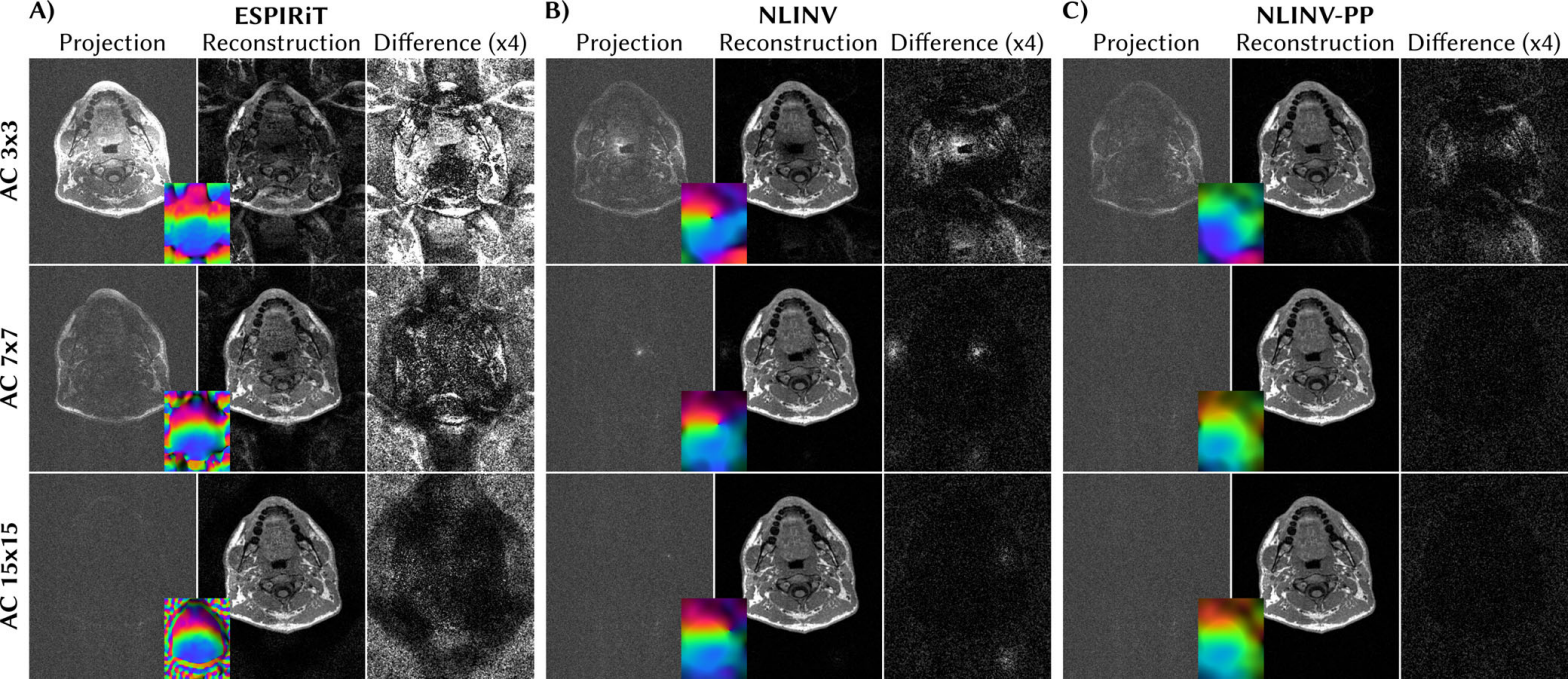
Evaluation of coils estimated with ESPIRiT (A), NLINV (B), and NLINV with phase pole correction (C). The first column shows the root‐sum‐of‐squares difference between the coil images and their point‐wise projection to the space spanned by the coil sensitivities. The second column shows reconstructed images using the respective coils in an $\ell_{1}$‐Wavelet reconstruction. The third column shows the difference of reconstructions to the fully sampled reference. Rows correspond to different sizes of full‐sampled AC regions. The inlaid images show the first estimated virtual coil to visualize the position of the phase pole (phase is color‐coded as in Figure 1). NLINV provides good coil sensitivities starting from 7×7 AC regions, while phase pole correction removes remaining artifacts in this example. ESPIRiT requires larger AC regions to achieve similar results.

We present the results of the projection tests, that is, the root‐sum‐of‐squares difference of the coil images and their projection to the space spanned by the respective coil sensitivities, in the first column of Figure 4A–C, respectively. Recall, ideal results only show noise and no image information. The projection for NLINV coil sensitivities does not show image structures from AC regions larger than 7×7, whereas ESPIRiT requires larger AC regions (15×15) to achieve similar results. The effect is also visible in the reconstructed images which show aliasing artifacts for ESPIRiT with small AC‐regions. The phase pole in the coil sensitivities obtained with NLINV without correction also leads to residual errors which are visible in the projection and the reconstructed image, and absent for NLINV with correction.

**FIGURE 5:**
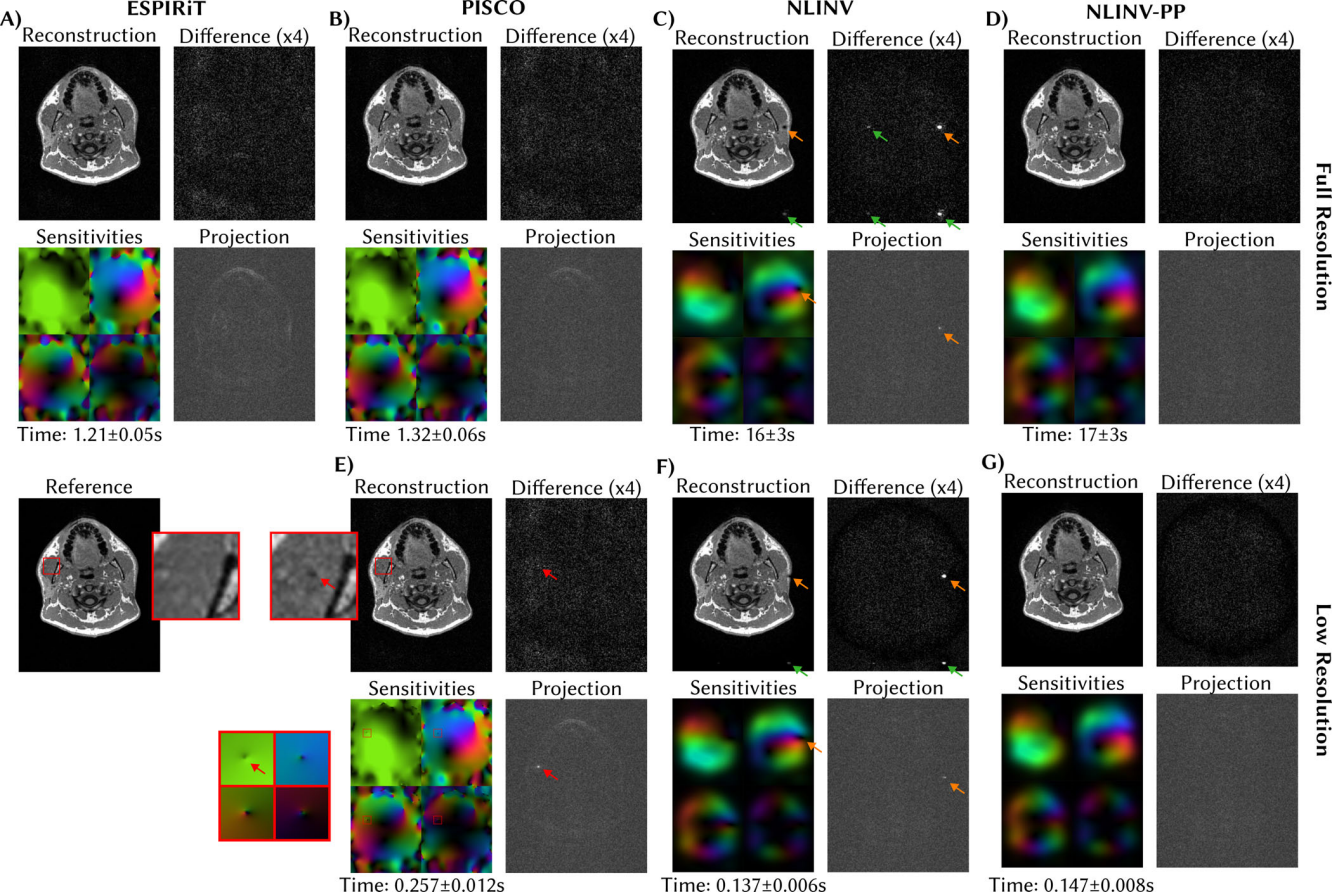
Comparison of different coil sensitivity estimation methods in terms of estimation time, projection error, and reconstruction error. In the top row (A–D), coil sensitivities (phase is color‐coded as in Figure 1) are estimated on the full resolution grid. ESPIRiT and PISCO are similarly fast, while NLINV is significantly slower. However, with phase pole correction, NLINV provides the best coil sensitivities in terms of the projection test, leading to the lowest difference of the reconstruction to the reference. Phase poles are marked by orange arrows and corresponding aliasing artifacts by green arrows. The bottom row (E–G) shows results for coil sensitivities estimated on a low‐resolution grid by PISCO and NLINV. Due to interpolation of a non‐smooth phase, PISCO introduces an artifact (red arrow) in the coils, which is visible in the projection test and leads to an artifact (dark spot visible in the inset) in the reconstruction.

Results of the measurement of coil sensitivity estimation time, are shown in Figure 5. It can be seen that both, PISCO and NLINV significantly benefit from use of a low‐resolution grid for coil sensitivity estimation. For PISCO, the interpolation of the coil sensitivities to the target resolution introduces an artifact in the coil sensitivities, probably due to non‐smooth phase variations, which is not visible in the high‐resolution estimation, but leads to an artifact in the final reconstruction. We saw similar artifacts in neighboring image slices, probably due to the non‐trivial phase in the jaw region. NLINV intrinsically provides smooth coil sensitivities that can be interpolated to the target resolution without visually worsening the results of the projection test compared to the full resolution estimation. The additional overhead for phase pole correction in NLINV is below 10% of the total estimation time.

**FIGURE 6:**
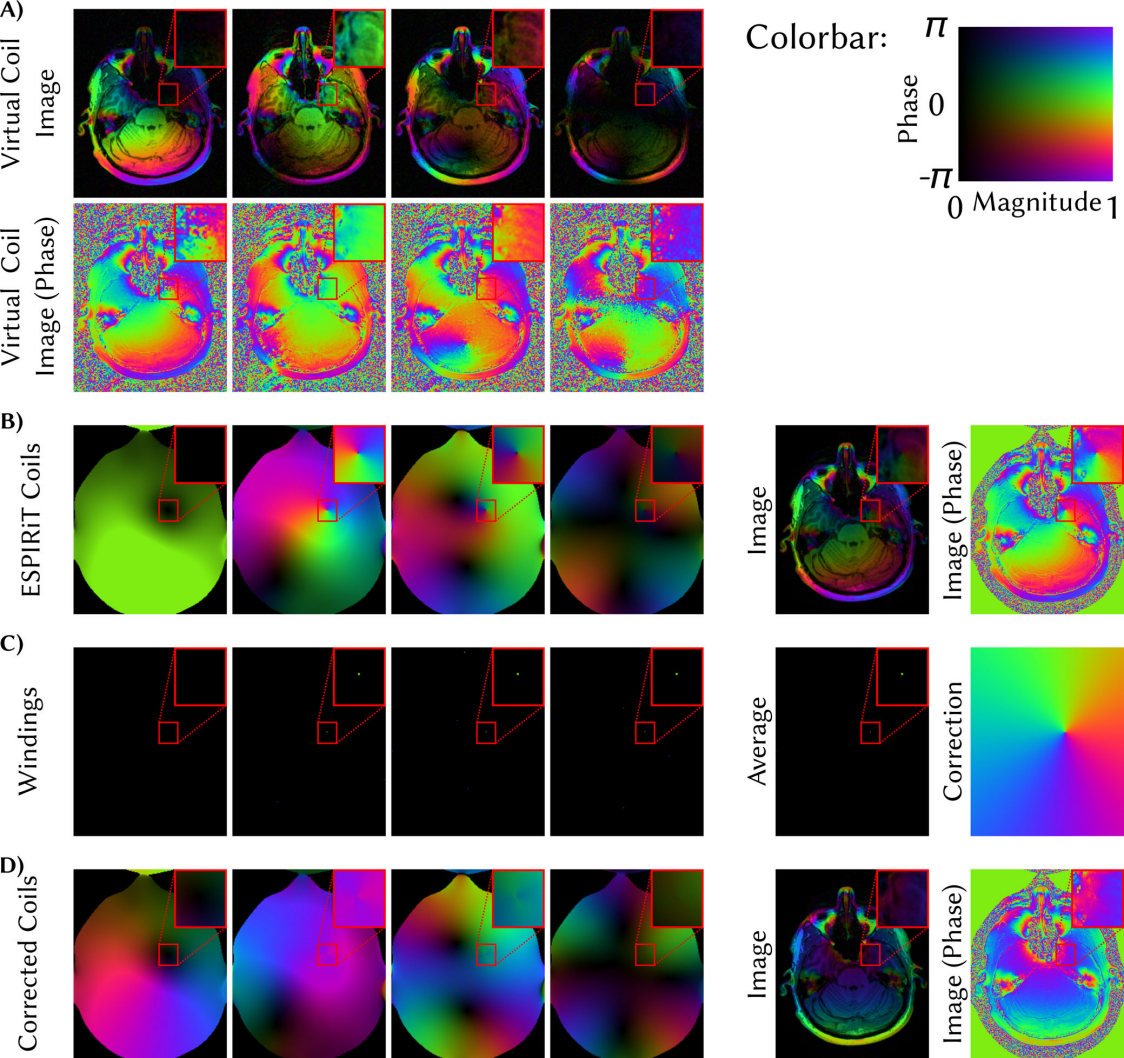
Application of the phase pole correction to ESPIRiT coils. (A) The first four coil images after coil compression. The first virtual coil image has a phase pole in the red square. (B) ESPIRiT coil sensitivities and coil‐combined image using these ESPIRiT coils. As the first virtual coil is used as reference (it has phase zero), the phase pole is also present in the coil combined image which has the phase of the first coil image. An opposite phase pole is visible in all other coil sensitivities to compensate the pole in the image. (C) Phase pole correction is performed on the coil sensitivities in (B). (D) Corrected coils and corresponding coil combined image. The phase pole is removed, however, the corrected phase in the coils is not perfectly smooth.

Phase pole correction for ESPIRiT coils is shown in Figure 6. Since ESPIRiT uses the first virtual coil as reference and this coil image has a phase pole visible in the inset of Figure 6A, the pole is also present in the coil combined image shown in Figure 6B. The correction yields a phase‐pole‐free coil combined image in Figure 6D, however, the phase in the corrected coils is not perfectly smooth.

**FIGURE 7:**
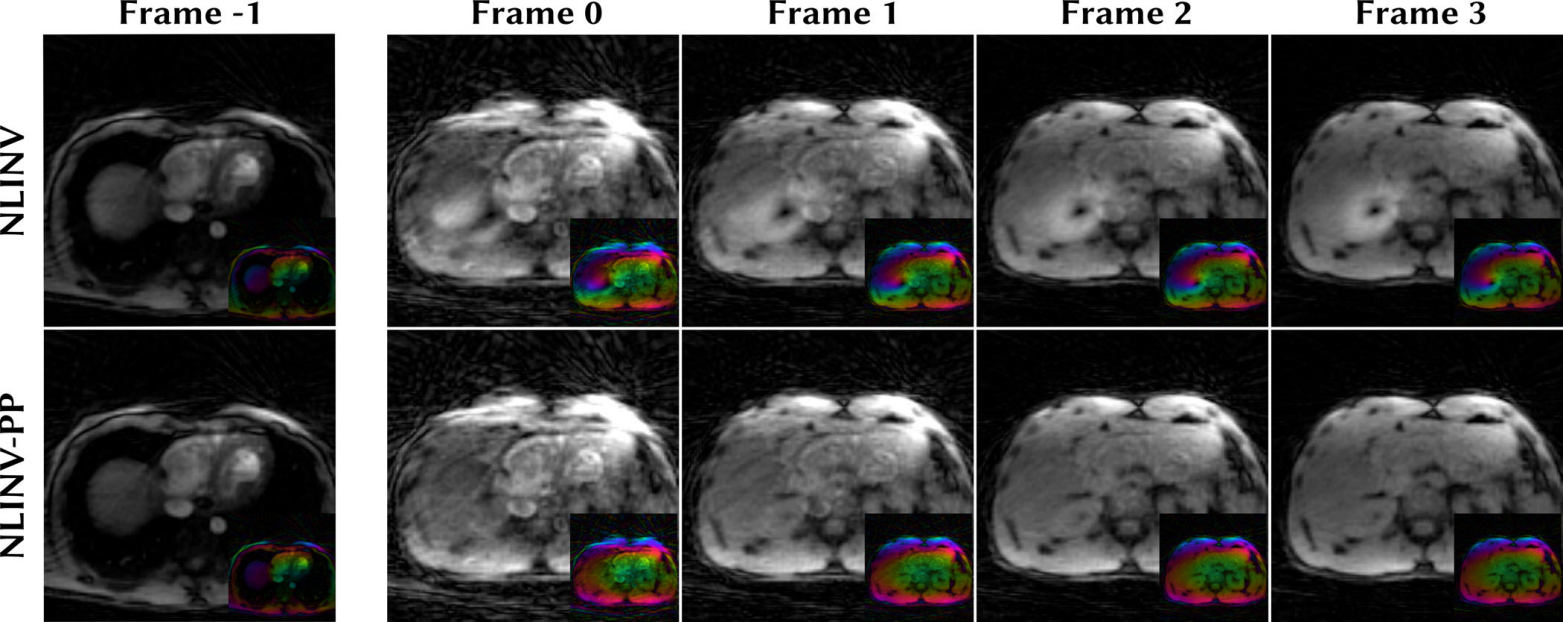
Real‐time reconstruction with (NLINV‐PP) and without (NLINV) phase pole correction. The imaging slice is interactively changed between Frame −1 and Frame 0 which in this example causes a phase pole to occur in the reconstruction. This pole is detected and corrected in the next frame (Frame 1). Insets show the complex‐valued images with phase color‐coded as in Figure 1.

Reconstructions of the real‐time data are shown in Figure 7. NLINV with phase pole correction detects and corrects the phase pole induced by the abrupt change of the imaging slice between Frame −1 and Frame 0. A movie of the full real‐time reconstruction is provided in the Supporting Information. Reconstruction of the whole time series takes 22s without phase pole correction and 25s with phase pole correction on an Nvidia H100 GPU. Both below the acquisition length of 32s, which is the limit for real‐time reconstruction.

## 4 Discussion

In this work, we present a simple method to obtain phase‐pole‐free images from NLINV reconstructions. Phase poles are detected by computing the winding numbers in the coil sensitivities. If the coils agree on a phase pole at the same location, it is considered an artificial phase pole. This technique is more robust compared to detecting phase poles directly in the image which is prone to noise in low signal intensity regions. Nevertheless, the technique requires three parameters to be set: the diameter d of circles used to compute the winding number, the threshold t for detecting phase poles, and the minimum distance $d_{closing}$ between detected phase poles to consider them as distinct. All our experiments worked well with the same default values of d=0.05FoV, t=0.5, and $d_{\text{closing}}=d$.

The current work is focussed on phase pole correction in NLINV. As shown in Figure 6, coil‐sensitivity‐based phase pole detections can also be applied to other coil sensitivity estimation methods such as ESPIRiT. However, the iterative approach of NLINV is particularly well suited for this task as the coil sensitivities can be refined after the correction in the following iterations leading to smooth sensitivities.

From a computational perspective, the method is efficient and has negligible, that is, below 10%, overhead compared to the NLINV reconstruction. NLINV itself could be optimized by alternative initialization strategies, for example, a low‐resolution NLINV reconstruction in the first Gauss‐Newton steps, which may have computational advantages in some scenarios. The computation of the winding numbers is trivially vectorized and can be efficiently performed on the GPU (if one is available). This allows us to utilize the method for interactive real‐time MRI applications which are prone to phase poles due to interactive changes of the imaging slice.

A current limitation of the method is its restriction to 2D reconstructions. In 3D, phase poles are not singular points but form lines around which the phase wraps [67], also called vortex lines. Hence, detection of 2D coordinates of phase poles in 2D turns to identification of 1D curves in 3D. If a vortex line is mostly orthogonal to the slices of a 3D volume, its intersection with the slice is a point and the 2D method can be applied slice‐by‐slice. However, this will likely fail if the vortex line is approximately parallel to the slices, as the singularity in the plane will be more similar to a phase jump than to a phase vortex. We plan to extend the method to 3D reconstructions in future work.

Finally, it should be noted that NLINV can also be used to estimate the coil sensitivity profiles for use in other reconstruction algorithms. We showed in this work, that NLINV can estimate high quality coil sensitivity profiles even from very small (7×7) AC regions. Compared to ESPIRiT or PISCO, which require larger AC regions, NLINV has the advantage that it can directly be applied to non‐Cartesian data, that it is computationally cheaper on a low‐resolution grid, and that it can be more easily accelerated with GPUs [75], motivating its use in several recent studies [32, 33, 34, 35, 36, 37, 38, 39, 40]. The remaining problem of the algorithm to get stuck in local minima with phase poles is solved by the proposed phase pole correction method.

## 5 Conclusion

As shown in previous studies, NLINV is an efficient and reliable tool for image reconstruction and coil sensitivity estimation in challenging applications. Nevertheless, in some applications, NLINV was still affected by phase pole related artifacts similar to ESPIRiT and other parallel imaging methods. In this work, we presented a method to detect and correct phase poles which solves this issue.

## Funding

This work was supported by NIH under grant U24EB029240, and by the Deutsche Forschungsgemeinschaft (DFG, German Research Foundation)—Project‐ID 432680300—SFB 1456, DZHK (German Centre for Cardiovascular Research) funding code: 81Z0300115. The research was funded in whole or in part by the Austrian Science Fund (FWF) Grant No. 10.55776/F100800.

## Conflicts of Interest

The authors declare no conflicts of interest.

## Supporting information




**Figure S1.** Phase pole detection at the example of a real dataset. The image, coil sensitivities and their product of the NLINV reconstruction in Figure 3 after 8 Gauss‐Newton steps are shown in phase (A) and magnitude + phase representation (B). The orange circles mark the position of the phase pole in the image, white circles mark the position of phase poles in the coil images. (C) illustrates the steps of the detection and (D) shows the final corrected images. The final images show the effect of changing specific steps of the algorithm. Namely, in (De) the circle diameter is reduced such that the poles detected in the respective coils do not overlap leading to no detected pole after the thresholding. In (Dc) and (Dd), the closing operation is skipped leading to two distinct detected poles. The final correction over‐compensates the pole present in the image leading to an opposite pole. For (Db) and (Dd), the global phase selection is skipped, leading to a generally larger change of the overall image phase. (Da) shows the final corrected image with all steps as presented in Figure 3.
**Video S1.** Real‐time reconstruction of the dataset shown in Figure 7 without (left) and with (right) phase pole correction.

## Data Availability

In the spirit of reproducible research, the code to reproduce the results of this paper is available at https://gitlab.tugraz.at/ibi/mrirecon/papers/phase‐pole (Version v0.2). All reconstructions have been performed with BART, available at https://github.com/mrirecon/bart. The data used in this study is available at Zenodo https://doi.org/10.5281/zenodo.16737746.

## ENDNOTES

^1^

https://github.com/ralobos/PISCO, Commit 986a00b.
