# Supplementary material for: Phase‐Pole‐Free Images and Smooth Coil Sensitivity Maps by Regularized Nonlinear Inversion
Source: Magn Reson Med. 2026 Mar 12;96(1):134–45. doi: 10.1002/mrm.70333 (PMC13156459; doi:10.1002/mrm.70333)
Supplement: Supplementary file 1 — Figure S1. Phase pole detection at the example of a real dataset. The image, coil sensitivities and their product of the NLINV reconstruction in Figure 3 after 8 Gauss‐Newton steps are shown in phase (A) and magnitude + phase representation (B). The orange circles mark the position of the phase pole in the image, white circles mark the position of phase poles in the coil images. (C) illustrates the steps of the detection and (D) shows the final corrected images. The final images show the effect of changing specific steps of the algorithm. Namely, in (De) the circle diameter is reduced such that the poles detected in the respective coils do not overlap leading to no detected pole after the thresholding. In (Dc) and (Dd), the closing operation is skipped leading to two distinct detected poles. The final correction over‐compensates the pole present in the image leading to an opposite pole. For (Db) and (Dd), the global phase selection is skipped, leading to a generally larger change of the overall image phase. (Da) shows the final corrected image with all steps as presented in Figure 3. Video S1. Real‐time reconstruction of the dataset shown in Figure 7 without (left) and with (right) phase pole correction. [file MRM-96-134-s001.zip › mrm70333-sup-0002-Supinfo.pdf]

Supporting Information

Video S1

Real-time reconstruction of the dataset shown in Figure 7 without (left) and with (right) phase pole correction.

Supporting Figures

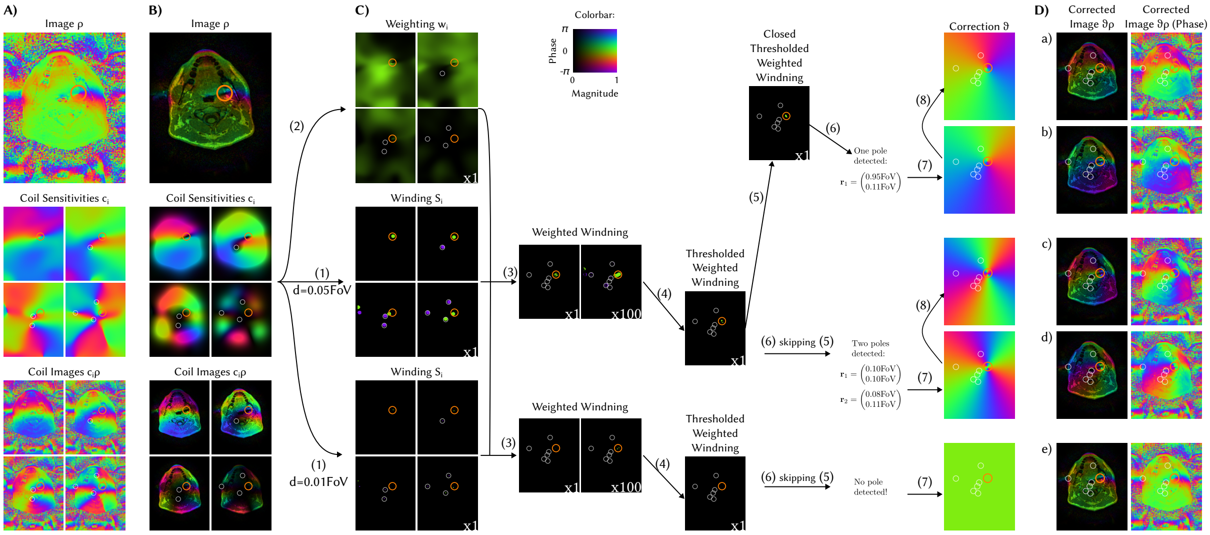

Figure S1: Phase pole detection at the example of a real dataset. The image, coil sensitivities and their product of the NLINV reconstruction in Figure 3 after 8 Gauss-Newton steps are shown in phase (A) and magnitude + phase representation (B). The orange circles mark the position of the phase pole in the image, white circles mark the position of phase poles in the coil images. (C) illustrates the steps of the detection and (D) shows the final corrected images. The final images show the effect of changing specific steps of the algorithm. Namely, in (De) the circle diameter is reduced such that the poles detected in the respective coils do not overlap leading to no detected pole after the thresholding. In (Dc) and (Dd), the closing operation is skipped leading to two distinct detected poles. The final correction over-compensates the pole present in the image leading to an opposite pole. For (Db) and (Dd), the global phase selection is skipped, leading to a generally larger change of the overall image phase. (Da) shows the final corrected image with all steps as presented in Figure 3.
